# Association between being breastfed and cardiovascular disease: a population cohort study of 320 249 participants

**DOI:** 10.1093/pubmed/fdad016

**Published:** 2023-03-02

**Authors:** Shinya Nakada, Frederick K Ho, Carlos Celis-Morales, Jill P Pell

**Affiliations:** School of Health and Wellbeing, University of Glasgow, Glasgow G12 8RZ, UK; School of Health and Wellbeing, University of Glasgow, Glasgow G12 8RZ, UK; School of Cardiovascular and Metabolic Health, University of Glasgow, Glasgow G12 8TA, UK; Human Performance Lab, Education, Physical Activity and Health Research Unit, University Católica del Maule, Talca 3466706, Chile; School of Health and Wellbeing, University of Glasgow, Glasgow G12 8RZ, UK

**Keywords:** Chronic disease, Food and nutrition, Public health

## Abstract

**Background:**

Being breastfed is associated with lower cardiovascular risk factors but, to date, no studies have demonstrated a protective effect on cardiovascular disease (CVD). This study aims to address the limitations of previous studies, specifically insufficient statistical power and residual confounding, to determine if such association exists.

**Methods:**

This is a population-based retrospective cohort study of 320 249 men and women aged 40–69 years. Breastfeeding status was self-reported. CVD and myocardial infarction (MI) events and deaths based via linkage to hospitalization and death records.

**Results:**

Overall, 28 469 (8.4%) participants experienced a CVD event and 5174 (1.6%) experienced an MI. Following adjustment for sociodemographic, lifestyle and early life confounders, breastfeeding was associated with a reduced risk of CVD events (HR 0.97, 95% CI 0.94–1.00, *P* = 0.041), CVD deaths (HR 0.91, 95% CI 0.84–0.98, *P* = 0.017), MI events (HR 0.93, 95% CI 0.87–0.99, *P* = 0.033) and MI deaths (HR 0.81, 95% CI 0.67–0.98, *P* = 0.026).

**Conclusions:**

Child health benefits of breastfeeding are well established. However, the benefits of breastfeeding may extend into later life reinforcing the need to encourage and support breastfeeding.

## Introduction

Cardiovascular disease (CVD) accounts for one-third of all deaths globally,^1^ with three-quarters of CVD deaths now occurring in developing countries.^2^^,^^3^ The global cost of CVD is predicted to increase from $863 billion in 2010 to $1044 billion in 2030.^4^ Risk factors for CVD operate across the life-course; for example, intra-uterine growth restriction is associated with CVD in later life.^5^

The benefits of breastfeeding to child health are well established, and include reduced mortality and morbidity from infectious, gastrointestinal and allergic diseases.^6–8^ There is evidence that breastfeeding is also associated with a number of CVD risk factors. A systematic review conducted in 2015 identified 38 studies of breastfeeding and systolic blood pressure and 46 studies of breastfeeding and cholesterol.^9^ Meta-analysis suggested that, among people who had been breastfed, systolic blood pressure was 0.80 mmHg (95% confidence interval [CI] 0.43–1.17) lower, and total cholesterol concentrations were 0.01 mmol/L (95% CI 0.02–0.05) lower. A meta-analysis published in 2019 identified 14 studies of breastfeeding and diabetes conducted on a total of 467 545 participants.^10^ The investigators derived a pooled odds ratio of 0.67 (95% CI 0.56–0.80) for breastfeeding. Finally, a meta-analysis in 2020 on breastfeeding and obesity pooled data on 332 297 participants across 25 studies to produce an odds ratio of 0.83 (95% CI 0.73–0.94) for breastfeeding.^11^

Randomized trial evidence on breast feeding is limited. Although a cluster randomized trial of advice to breast-feed healthy term infants reported no effect on adiposity and blood pressure at six months of age,^12^ randomization of individual preterm infants to receive banked breast milk or preterm formula milk produced significant differences in blood pressure,^13^ lipid profile,^14^ and leptin resistance^15^ at 13–15 years of age, with evidence of a dose–response relationship. There is also evidence of an association between breast feeding and atherosclerosis. Sixty five year follow-up of 405 participants in the Boyd Orr cohort demonstrated inverse associations between breastfeeding and carotid intima-media thickness, carotid plaques and femoral plaques.^16^

In spite of evidence of associations with CVD risk factors and sub-clinical atherosclerosis, there is currently no evidence that breastfeeding reduces the risk of CVD itself. A meta-analysis of four studies on a total of 14 792 subjects reported no association with cardiovascular mortality (pooled rate ratio 1.06, 95% CI 0.94–1.20),^17^ and a separate study of 87 252 women produced non-significant results for both coronary heart disease (hazard ratio [HR] 0.92, 95% CI 0.80–1.05) and stroke (HR 0.91, 95% CI 0.79–1.06).^18^ The aim of this study is to determine whether breastfeeding is associated with CVD in middle and old age in a single, very large, prospective, general population cohort study.

## Methods

### UK Biobank

Between 2006 and 2010, UK Biobank recruited 502 487 participants (response rate of 5.5%), aged from 40 to 69 years, from the UK general population. Participants attended baseline assessment at one of 22 centres set up across England, Scotland and Wales. Data were collected via a self-completed touch-screen questionnaire, interviewer-completed questionnaire, physical and functional measurements, and sample collections of blood, urine and saliva. Follow-up was achieved via individual-level record linkage to routine databases covering primary care records, hospitalizations and deaths.

### Exposure and outcomes

In the self-completed baseline questionnaire, participants reported whether they had been breastfed as a baby or not. Four outcomes were studied: CVD events (including fatal and non-fatal events), CVD deaths, myocardial infarction (MI) events (including fatal and non-fatal events) and MI deaths. Events were ascertained from hospitalizations and deaths. CVD was defined as an International Classification of Diseases 10th revision (ICD-10) code of I11.0, I20-I25.9, I42.0, I42.7, I42.9, I50-I50.9, I60-I64 or I70-I173.9, and MI was defined as I21. Death data were provided by NHS Digital for England and Wales, and the NHS Central Register for Scotland. Hospitalization data were obtained from the Hospital Episode Statistics for England, the Patient Episode Database for Wales, and the Scottish Morbidity Record for Scotland. At the time of analysis, death data were available up to 1 June 2020. For mortality outcomes, follow-up was, therefore, censored at this date or date of death if this occurred earlier. Hospital admission data were available until 31 March 2017 for Scotland and Wales and 1 June 2020 for England. Therefore, for event outcomes, the follow-up was censored at these dates unless a relevant hospitalization or death from any cause preceded this.

### Covariates

Covariates were identified a priori based on existing evidence of associations with infant feeding method and CVD risk. The 13 potential confounder variables comprised: five sociodemographic factors (age, sex, ethnic group, income and area-based deprivation); three early life factors (birthweight, multiple birth and maternal smoking); and five lifestyle factors (smoking status, physical activity, and consumption of oily fish, red and processed meat). Age was derived from dates of birth and baseline assessment, and area-based deprivation was derived from postcode of residence. All other variables were self-reported at baseline using the touch-screen questionnaire. Age was categorized into: < 45, 45–49, 50–54, 55–59, 60–64 and ≥ 65 years. Income was categorized into: <£18 000, £18 000–£30 999, £31 000–£51 999, £52 000–£100 000 and > £100 000. Area-based deprivation was ascertained using the Townsend deprivation index which is derived from Census data on unemployment, car and home ownership, and overcrowding, and was categorized into quartiles. Birthweight was categorized into: < 2500, 2500–3999 and ≥ 4000 g. Smoking status was categorized as never, former and current smoker. Frequency and duration of walking, moderate and vigorous activity were self-reported using the International Physical Activity Questionnaire (IPAQ) short form. These were combined using the IPAQ scoring protocol to derive metabolic equivalents (MET-h/week) that were categorized into < 300, 300–599 and ≥ 600 MET-h/week. Self-reported frequency of consumption of oily fish, processed meat and red (beef, pork or lamb) meat were each categorized as low (never or less than weekly), medium (one to four times per week) and high (≥5 times per week).

The other covariates comprised of the following: systolic blood pressure (SBP); low density lipoprotein (LDL) cholesterol; triglycerides; diabetes; and body mass index (BMI). SBP was measured by research nurses at the baseline assessment using an automated Omron device 0-255. The average of two readings was taken and treated as a continuous variable. LDL cholesterol and triglycerides were measured on baseline blood samples using a Beckman Coulter AU5400 or 5800, and were treated as continuous variables. Diabetes was based on self-report of a physician diagnosis at baseline. Bodyweight and height were measured at baseline, after removal of shoes and socks, using a Tanita BC-418MA body composition analyser and SECA 240 height measure, respectively. BMI was derived and categorized as follows: underweight (BMI < 18.5 kg/m^2^), normal weight (BMI 18.5–24.9 kg/m^2^), overweight (BMI 25–29.9 kg/m^2^) and obese (BMI ≥ 30 kg/m^2^) in line with WHO recommendations.

### Statistical analyses

Participants were excluded if they did not provide information on their feeding method as an infant, they reported a history of CVD at baseline, did not provide information on their past medical history, or had missing data for continuous covariates. Participant characteristics were reported according to whether they had been breastfed or not using frequency and percentage for categorical data, mean and standard deviation (SD) for normally distributed continuous data and median and interquartile range (IQR) for skewed continuous data. The two groups were compared using Pearson chi-squared tests for binary and nominal variables, chi-square tests for trend for ordinal data, *t*-tests for normally distributed continuous data and Wilcoxon rank-sum tests for skewed continuous data. For categorical data, missing data were included as a missing category. Cox proportional hazard models were used to estimate the association between mode of infant feeding and outcomes. Statistical significance was defined as the 5% level. The proportional hazards assumption was checked using Schoenfeld residuals. For each outcome, five models were run: univariate; adjusted for sociodemographic confounders; adjusted for sociodemographic and lifestyle confounders; adjusted for sociodemographic, lifestyle and early life confounders; adjusted for all confounding plus potential mediators (SBP, LDL cholesterol, triglycerides, diabetes and BMI). S.N. and F.K.H. conducted all of the analyses using R version 4.0.3.

### Ethical consideration

UK Biobank was approved by the North-West Multi-Centre Research Ethics Committee (Ref: 11/NW/0382). The investigation conforms to the principles outlined in the Declaration of Helsinki. Informed consent was obtained from all individual participants included in the study. The study protocol is available online (http://www.ukbiobank.ac.uk/). This work was conducted under the UK Biobank application number 7155.

## Results

Of the 502 487 UK Biobank participants, 182 238 were excluded: 118 779 because they did not provide information on their feeding mode in infancy, 19 620 because they had a history of CVD of baseline, 815 because they did not provide information on their past medical history and 43 024 because they had missing values for continuous covariates. Consequently, a total of 320 249 participants were included in this study (Fig. S1).

Participants who reported having been breastfed were older, more physically active, more likely to be female and more likely to live in deprived areas compared with participants who reported not having been breastfed (Table 1). They were also less likely to have been low birthweight or part of a multiple birth, and less likely to be white. Their mothers were less likely to have smoked, and whilst they were more likely to have smoked at some point (43.8% vs 41.9%), they were less likely to be a current smoker (9.7% vs 11.1%). Finally, whilst they were more likely to eat oily fish and less likely eat processed meat, they were more likely to eat red meat. Participants who reported having been breastfed had a higher prevalence of cardiovascular risk factors at baseline: they were more likely to have diabetes, more likely to be overweight or obese, and their LDL cholesterol and triglyceride concentrations were higher (Table 1).

**Table 1:** Baseline characteristics and cardiovascular risk factors of participants according to whether they were breastfed or not

		Breastfed *N* = 232 174	Not breastfed *N* = 88 075	*P*-value
		*N* (%)	*N* (%)	
Sex	Female	131 603 (56.7)	55 544 (63.1)	<0.001
	Male	100 571 (43.3)	32 531 (36.9)	
Age (years)	<45	21 475 (9.2)	17 264 (19.6)	<0.001
	45–49	30 519 (13.1)	17 170 (19.5)	
	50–54	37 845 (16.3)	15 180 (17.2)	
	55–59	45 153 (19.4)	14 092 (16.0)	
	60–64	56 232 (24.2)	15 340 (17.4)	
	≥65	40 950 (17.6)	9029(10.3)	
Ethnicity	White	214 359 (92.3)	85 833 (97.5)	<0.001
	South Asian	6420 (2.8)	517 (0.6)	
	Black	5535 (2.4)	301 (0.3)	
	Chinese	801 (0.3)	266 (0.3)	
	Any other	2940 (1.3)	353 (0.4)	
	Mixed	1379 (0.6)	599 (0.7)	
Income (£)	≤18 000	41 002 (17.7)	14 770 (16.8)	<0.001
	18 000–30 999	49 870 (21.5)	18 190 (20.7)	
	31 000–51 999	53 449 (23.0)	21 865 (24.8)	
	52 000–100 000	44 364 (19.1)	17 684 (20.1)	
	≥100 000	12 545 (5.4)	4560 (5.2)	
Area-based deprivation quartile	Lowest	59 923 (25.8)	21 788 (24.8)	<0.001
	2	59 039 (25.5)	22 057 (25.1)	
	3	58 318 (25.1)	22 329 (25.4)	
	Highest	54 622 (23.6)	21 791 (24.8)	
Birth weight (g)	<2500	11 287 (7.7)	8299 (13.2)	<0.001
	2500–3999	114 395 (78.2)	47 157 (75.2)	
	4000–7000	20 525 (14.0)	7234 (11.5)	
Multiple birth	No	227 042(97.8)	82 884 (94.1)	<0.001
	Yes	4198 (1.8)	3100 (3.5)	
Maternal smoking	No	156 318 (67.3)	50 103 (56.9)	<0.001
	Yes	52 304 (22.5)	27 411 (31.1)	
Smoking	Never	129 732 (55.9)	50 937 (57.8)	<0.001
	Former	79 266 (34.1)	27 155 (30.8)	
	Current	22 458 (9.7)	9743 (11.1)	
Oily fish intake	Low	98 098 (42.5)	41 534 (47.4)	<0.001
	Middle	130 563 (56.5)	45 424 (51.8)	
	Hight	2350 (1.0)	704 (0.8)	
Processed meat intake	Low	96 021 (41.4)	35 146 (40.0)	<0.001
	Middle	127 085 (54.8)	49 474 (56.3)	
	High	8595 (3.7)	3319 (3.8)	
Red meat intake	Low	116 743 (50.3)	45 706 (51.9)	<0.001
	Middle	42 223 (18.2)	16 508 (18.7)	
	High	73 208 (31.5)	25 861 (29.4)	
Physical activity (MET-minutes/week)	<300	16 074 (8.4)	6625 (9.2)	<0.001
	300–599	18 184 (9.5)	6983 (9.7)	
	≥600	157 642 (82.1)	58 209 (81.1)	
Diabetes	No	221 232 (95.5)	84 805 (96.5)	<0.001
	Yes	10 414 (4.5)	3080 (3.5)	
BMI category	Underweight	1179 (0.5)	549 (0.6)	0.005
	Normal	78 972 (34.1)	31 028 (35.3)	
	Overweight	98 214 (42.4)	35 598 (40.5)	
	Obese	53 132 (23.0)	20 680 (23.5)	
		Mean (SD)	Mean (SD)	
SBP (mmHg)		137.7 (18.6)	135.3 (18.3)	<0.001
LDL cholesterol (mol/L)		3.61 (0.85)	3.58 (0.84)	<0.001
		Median (IQR)	Median (IQR)	
TG (mol/L)		1.46 (1.074)	1.42 (1.087)	<0.001

*N*, number; BMI, body mass index; SD, standard deviation; SBP, systolic blood pressure; LDL, low density lipoprotein; IQR, inter-quartile range; TG, triglyceride

For CVD events, there was a mean 10.6 years of follow-up, equating to a total of 3.4 million person-years of follow-up data. For deaths, the respective figures were 11.6 years and 3.7 million person-years. Overall, 28 469 (8.9%) participants experienced a CVD event equivalent to a crude annual incidence of 84/10 000 participants; 21 804 (9.4%) of those who were breastfed and 6 665 (7.6%) of those who were not (89 vs 71/10 000/pa) (Table 2).

**Table 2:** Crude CVD and MI incidence and mortality rates by infant feeding mode

	Breastfed			Not breastfed			Overall		
**Events**	***N***	**%**	**Incidence/10 000/pa**	***N***	**%**	**Incidence/10 000/pa**	***N***	**%**	**Incidence/10 000/pa**
CVD	21 804	9.4	89	6665	7.6	71	28 469	8.9	84
MI	3930	1.7	15	1244	1.4	13	5174	1.6	15
**Deaths**	**N**	**%**	**Mortality/10 000/pa**	**N**	**%**	**Mortality/10 000/pa**	**N**	**%**	**Mortality/10 000/pa**
CVD	2749	1.2	10	831	0.9	8	3580	1.1	10
MI	462	0.2	2	155	0.2	2	617	0.2	2

*N*, number; pa, per annum; CVD, cardiovascular disease; MI, myocardial infarction

Overall, 5174 (1.6%) participants experienced an MI event equivalent to a crude annual incidence of 15/10 000 participants; 3930 (1.7%) of those who were breastfed and 1244 (1.4%) of those who were not (15 vs 13/10 000/pa).

**Fig. 1:**
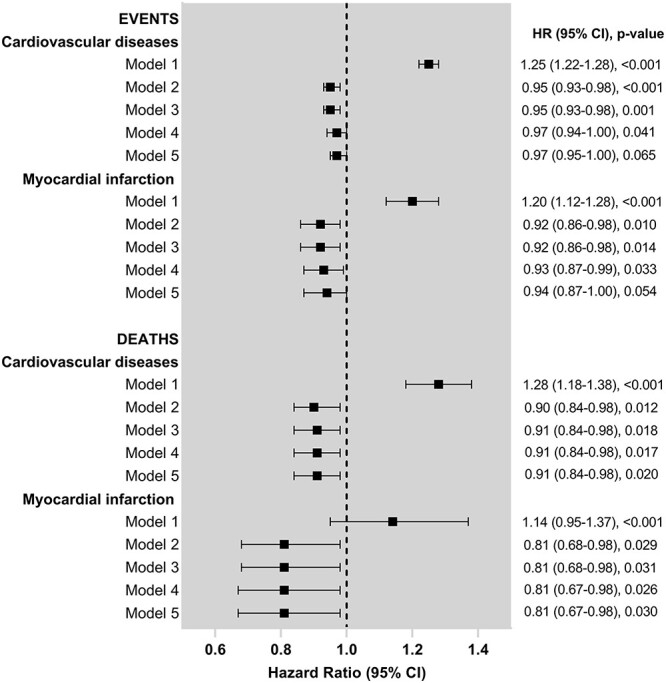
Cox-proportional hazard models of the association between breastfeeding and CVD and MI events and deaths. HR, hazard ratio; CI, confidence interval.

In the univariate analysis, people who were breastfed were more likely to experience CVD events. After adjustment for sociodemographic factors, the association remained significant, but the direction was reversed, with people who were breastfed less likely to experience CVD events. The association remained statistically significant after adjustment for lifestyle and early life factors (Table 3, Fig. 1). A similar pattern was observed for CVD deaths and MI events. It was also similar for MI deaths other than the univariate association not reaching statistical significance. When the potential mediators were added to the models, the associations with CVD and MI events became non-significant but there was no meaningful change in any of the effect size estimates.

**Table 3:** Cox-proportional hazard models of the association between breastfeeding and CVD and MI events and deaths

	Model 1 *n* = 320 249		Model 2 *n* = 320 249		Model 3 *n* = 320 249		Model 4 *n* = 320 249		Model 5 *n* = 320 249	
	HR (95% CI)	*P*-value	HR (95% CI)	*P*-value	HR (95% CI)	*P*-value	HR (95% CI)	*P*-value	HR (95% CI)	*P*-value
Events										
CVD	1.25 (1.22–1.28)	<0.001	0.95 (0.93–0.98)	<0.001	0.95 (0.93–0.98)	0.001	0.97 (0.94–1.00)	0.041	0.97 (0.95–1.00)	0.065
MI	1.20 (1.12–1.28)	<0.001	0.92 (0.86–0.98)	0.010	0.92 (0.86–0.98)	0.014	0.93 (0.87–0.99)	0.033	0.94 (0.87–1.00)	0.054
Deaths										
CVD	1.28 (1.18–1.38)	<0.001	0.90 (0.84–0.98)	0.012	0.91 (0.84–0.98)	0.018	0.91 (0.84–0.98)	0.017	0.91 (0.84–0.98)	0.020
MI	1.14 (0.95–1.37)	0.152	0.81 (0.68–0.98)	0.029	0.81 (0.68–0.98)	0.031	0.81 (0.67–0.98)	0.026	0.81 (0.67–0.98)	0.030

Model 1: no adjustment

Model 2: adjusted for sociodemographic (age, sex, ethnicity, income, area-deprivation) confounders

Model 3: adjusted for sociodemographic and lifestyle (smoking status, physical activity, oily fish, processed meat, red meat) confounders

Model 4: adjusted for sociodemographic, lifestyle and early life (birth weight, multiple birth, maternal smoking) confounders

Model 5: adjusted for sociodemographic, lifestyle, early life confounders and systolic blood pressure, low-density lipoprotein cholesterol, triglycerides, diabetes, body mass index

n, number; HR, hazard ratio; CI, confidence interval; CVD, cardiovascular disease; MI, myocardial infarction

## Discussion

### Main finding of this study

Participants who had been breastfed were at higher risk of CVD and MI univariately. However, this was explained by sociodemographic confounding. After adjusting for this, the associations were reversed and there was evidence that breastfeeding had a protective association with fatal and non-fatal CVD and MI events in middle- and older-age.

The univariate results are not surprising, given historical trends in breastfeeding. UK Biobank participants were born between 1937 and 1970. From the 1900s to the 1960s, breastfeeding rates in the Western world declined because of the emergence of alternative feeding methods and changes in lifestyle. During this period, breastfeeding was mainly practiced by less affluent women.^19^^,^^20^ From the 1960s onward, there has been a gradual resurgence in breastfeeding led by affluent and better-educated women because of a better appreciation of its health benefits.^19^^,^^20^ Therefore, more deprived UK Biobank participants were more likely to have been breastfed, as well as being more likely to develop CVD.

### What is already known in this topic

Whilst associations demonstrated in observational studies are not, necessarily, causal, a causal association between breastfeeding and CVD is biologically plausible. The high cholesterol content of breast milk reduces cholesterol synthesis by down-regulating hepatic hydroxymethylglutaryl coenzyme A (HMG-CoA).^21^ Consumption of breast milk may protect against the development of obesity because of lower protein intake,^22^ lower insulin release,^23^ or different future dietary preferences.^24^ Breastfeeding increases long-chain polyunsaturated fatty acids resulting in lower glucose concentrations, protecting against insulin resistance^25^ and reducing blood pressure.^26^ However, in our study, adding SBP, LDL cholesterol, triglycerides, diabetes and BMI did not substantially attenuate the association between being breastfed and CVD, providing limited evidence that these are the underlying mechanism.

Our study is the largest study to investigate the association between breastfeeding and CVD and the first to report a significant, protective association. A meta-analysis of four studies comprising a total of 14 792 subjects born between 1904 and 1939 derived a pooled rate ratio of 1.06 (95% CI 0.94–1.20, *P* = 0.3).^17^ Subsequent to its inclusion in the systematic review, an updated analysis of the Caerphilly cohort suggested that, among 1602 men, those who had been breastfed were at increased risk of CHD mortality (HR 1.73, 95% CI 1.17–2.55) and incidence (HR 1.54, 95% CI 1.17–2.04) but the authors attributed this counterintiuitive result to either information or selection bias.^27^ Finally, an analysis of 87 252 female nurses born between 1921 and 1946 demonstrated a non-significant negative association between breastfeeding and CVD cases (HR 0.91, 95% CI 0.83–1.01).^18^ The inconsist findings reported by previous studies could be due to smaller sample sizes in some and failure to adjust for important confounders, such as socioeconomic status and ethnic group.

### What this study adds

The WHO set a target of 50% of infants being exclusively breastfed in the first six months of life based on well-established evidence of short-term health benefits. Our study provides evidence that the benefits of breastfeeding may, in fact, extend over many decades. Rates of breastfeeding increased by around 2.4% per annum between 1985 and 1995 but have since declined in many regions.^28^ Currently, only 38% of infants globally are exclusively breastfed up to 6 months of age, well short of the WHO target. Our study reinforces the importance of encouraging and supporting breastfeeding.

### Limitation of this study

UK Biobank is a very large, prospective, general population cohort that provided data on both fatal and non-fatal outcomes. Data were available on a large range of potential confounders but dietary intake and physical activity were measured at a single time-point and may have changed over time. Also, in spite of adjusting for multiple confounders, residual confounding due to unmeasured or unknown confounders is a potential weakness of any observational study. Mode of infant feeding was self-reported but obtained prior to the occurrence of the outcome making systematic errors in reporting unlikely. UK Biobank is not representative of the general population in terms of lifestyle. Therefore, care should be taken in generalizing summary statistics and absolute rates. However, estimates of effect size should be generalizable. The results of our study are valid for comparisons with formula milk used between 1937 and 1970. However, the composition of formula milk has changed over time and is now closer to breast milk.^29^ Therefore, this study should be repeated in the future to determine if the results remain valid for contemporary formulations. It should also be noted that there could be survival bias underestimating the current association, as breastfed infants were less likely to have infection and more likely to survive and being recruited into the UK Biobank. A limitation of our study was the lack of data on mixed feeding and duration of breastfeeding, which would have enabled investigation of a possible dose relationship.^30^ Lastly, missing data were assumed to be missing completely at random, which might not be true. However, this is unlikely to alter our conclusion, given the relatively small proportion of missingness.^31^

## Supplementary Material

Figure_S1_fdad016

Figure_S2_Legend_fdad016

## Data Availability

This study used data from the UK Biobank cohort under a data sharing agreement (reference 7155). Under the terms of that agreement, we are not permitted to share the data with a third party. Anyone wishing to access UK Biobank data should contact UK Biobank directly via https://www.ukbiobank.ac.uk/.

## Notes

**Shinya Nakada**, MPH

**Frederick K. Ho**, Lecturer in Public Health

**Carlos Celis-Morales**, Research Fellow

**Jill P. Pell**, Director of the Institute of Health and Wellbeing and Henry Mechan Professor of Public Health
