# Supplementary figures and images for: Association between being breastfed and cardiovascular disease: a population cohort study of 320 249 participants
Source: J Public Health (Oxf). 2023 Mar 2;45(3):569–76. doi: 10.1093/pubmed/fdad016 (PMC10470327; doi:10.1093/pubmed/fdad016)

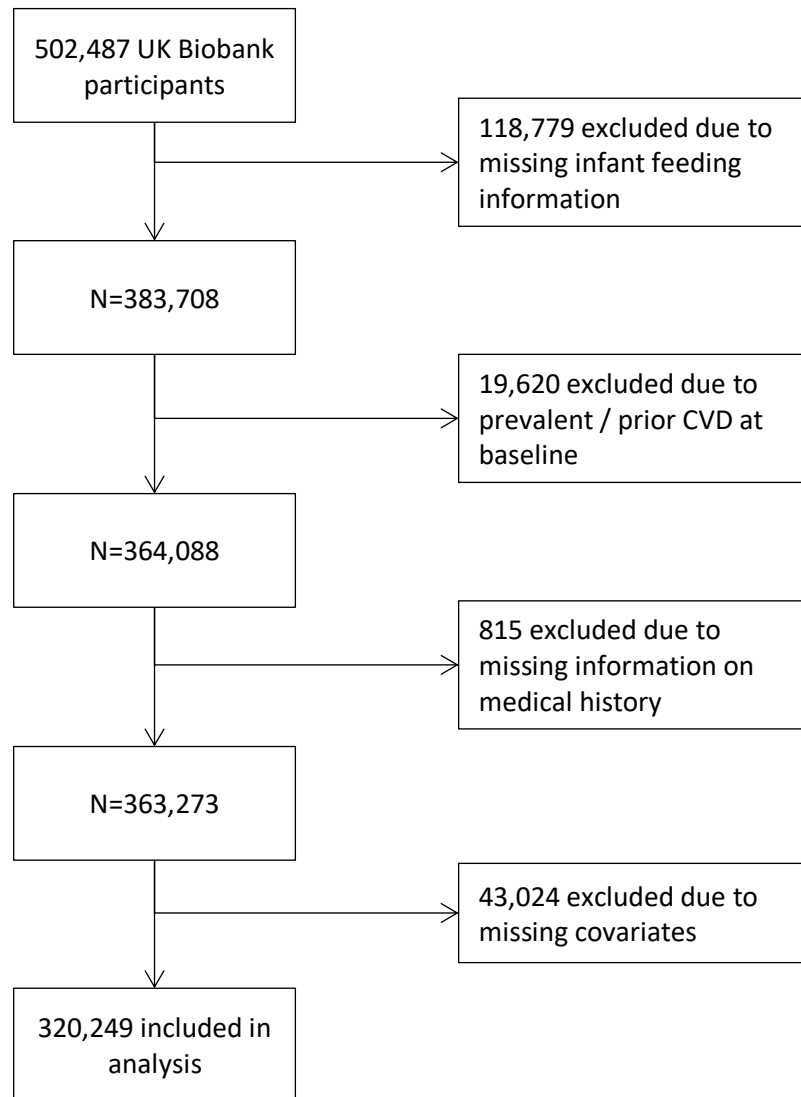

Supplement: Figure_S1_fdad016 [file figure_s1_fdad016.pdf]
